# Gnotobiotic evaluation of *Dalbergia sissoo* genotypes for resistance against *Fusarium solani* via dual culture set up

**DOI:** 10.1186/s43141-021-00132-3

**Published:** 2021-02-23

**Authors:** Monika Chauhan, Ajay Thakur, Yashaswi Singh, M. Maqbool Rather, Nirmal S. K. Harsh

**Affiliations:** 1 Tissue Culture Laboratory, Genetics and Tree Improvement Division, Forest Research Institute, Dehra Dun, Uttarakhand 248006 India; 2 Faculty of Forestry, SKUAST-Kashmir, Benehama, Ganderbal, Jammu and Kashmir India; 3 Forest Pathology Division, Forest Research Institute, Dehra Dun, Uttarakhand 248006 India

**Keywords:** *Fusarium solani* f. sp. *dalbergiae*, *Fusarium* wilt, Genetic variation, Gnotobiota, In vitro screening, Shisham mortality, Plant-microbe interaction

## Abstract

**Background:**

*Dalbergia sissoo* (shisham), an important multipurpose tree native to the Indian subcontinent and also planted in other countries, has been afflicted with large scale mortality in all age groups due to wilt disease, causing huge economic losses. *Fusarium solani* f. sp. *dalbergiae* (*Fsd*) has been identified as one of the causal organisms for wilt disease in *D. sissoo*. One of the approaches of disease resistance studies involves co-cultivation of trees and pathogens under controlled conditions to screen resistant tree genotypes. A gnotobiotic condition, where the pathogen is known, enables accurate screening of disease-resistant genotypes. In the present study, ten genotypes of *D. sissoo* were cloned in vitro and evaluated against two strains of *Fsd* in a dual culture setup under gnotobiotic conditions with an objective to identify resistant genotypes of *D. sissoo* against *Fsd*.

**Results:**

Callus and plantlets of ten genotypes of host plant multiplied in vitro were inoculated with conidial suspension of two strains of *Fsd* at three concentrations; 1 × 10^1^, 1 × 10^3^, and 1 × 10^5^ conidia/ml. Gnotobiotic evaluation of dual culture setup shows variations among genotypes in their response towards in vitro *Fsd* infection; and two genotypes (14 and 66) exhibited resistance against *Fsd* strains. Callus of genotypes 14 and 66 significantly restricted the fungal mycelium growth whereas callus of remaining genotypes was completely infested by *Fsd* mycelium within 9 days. Similarly, plantlets of genotype 14 and 66 had lesser disease severity and remained green and had fewer necrotic lesions in roots whereas plantlets of the remaining eight genotypes died within 15 days.

**Conclusion:**

Gnotobiotic evaluation of callus and plantlets of ten genotypes of *D. sissoo* against *Fsd* strains has reduced time and space otherwise required for field trials. Genetic variations amongst the genotypes resulted in varying responses towards virulent *Fsd* strains and only two out of ten genotypes showed promising resistant characteristics. In dual culture setup, both callus and plantlets of the same genotypes responded similarly against *Fsd* strains, which signify that in vitro screening can be used as an indirect selection method for disease resistance.

## Background

*Dalbergia sissoo* Roxb. ex DC., commonly known as shisham, is a valuable timber species in Fabaceae family, native to Afghanistan, Bangladesh, Bhutan, India, Iraq, Iran, Myanmar, Nepal, and Pakistan and introduced into Africa, Australia, China, and the USA [1–3]. It is a pioneer tree of primary succession in natural riverine forest of rivers Indus, the Ganges, Yamuna and Brahmaputra with their tributaries and also an important multipurpose tree growing outside forest which yields beautiful dark brown wood for furniture and panels, additionally used for strong poles, quality fodder, fuel wood and folk medicine [4]. The species is a suitable tree for wheat-based agroforestry system as it is deciduous and fixes nitrogen [5, 6].

**Fig. 1:**
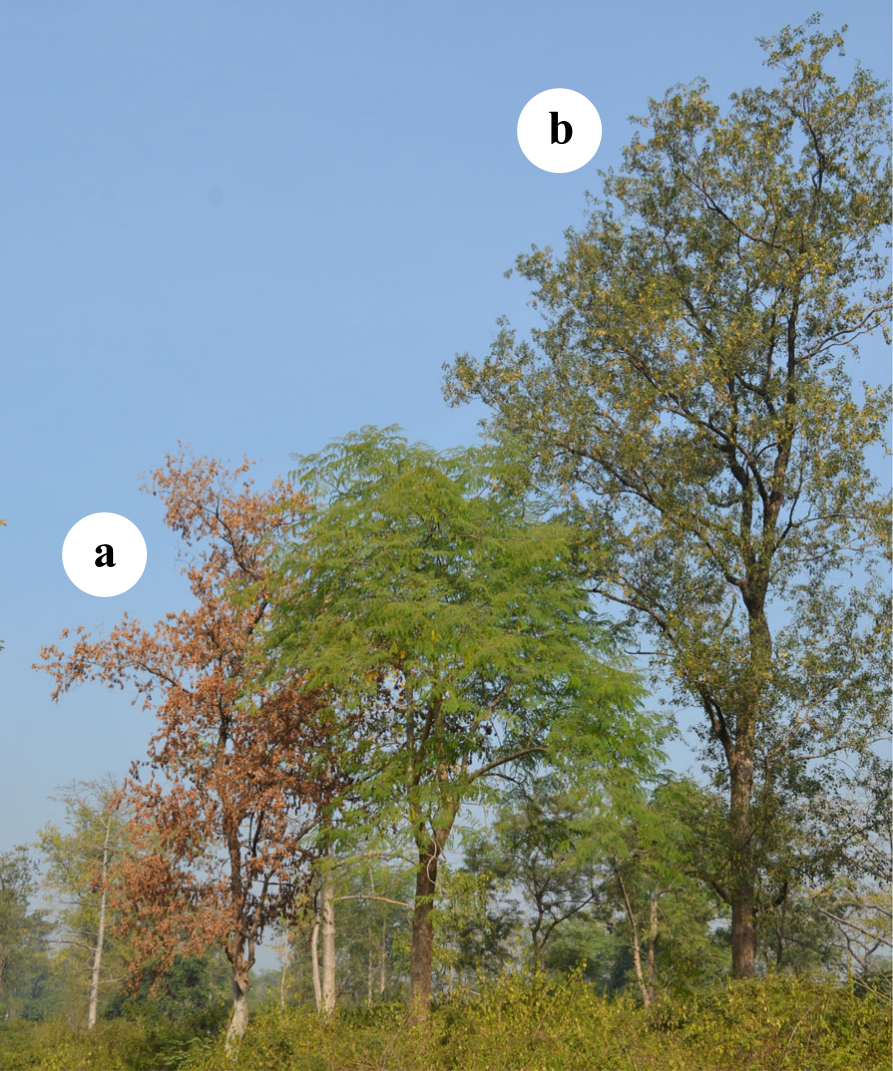
*D. sissoo* completely wilted (a) and healthy (b) trees in their natural habitat (riverine forest) Thano forest range, Dehra Dun, India

Widespread mortality in *D. sissoo* (Fig. 1) has been reported from many parts of India [7–14] as well as from Bangladesh [15], Nepal [16], and Pakistan [17], and one study in India estimated a loss equivalent to US$ 200 million due to mortality of 400,000 mature trees [12, 18]. *Fusarium solani* (Mart.) Sacc. f. sp. *dalbergiae* Bakshi and Singh (hereafter referred as *Fsd*) has been reported as one of the primary pathogenic fungi causing mortality in *D. sissoo* due to vascular wilt which is prevalent in the riverine plains of India, Pakistan, Nepal, and Bangladesh [8]. Other fungal pathogens viz., *Fusarium oxysporum*, *Oxyporus latemarginatus* [19], *Ganoderma lucidum* (Curtis) P. Karst [20], *Cercospora sissoo* [21], *Colletogloeum sissoo*, *Glomerella cingulata* and *Septothyrella dalbergiae* [22], *Mycosphaerella dalbergiae* [23], *Phyllachora dalbergiae* (=*Phyllachora viventis*; [24]), *Phyllactinia dalbergiae* [25], *Maravalia achroa* and *Uredo sissoo* [26], *Ceratocystis manginecans* M. Van Wyk, Al Adawi & M. J. Wingf [27], and *Lasiodiplodia theobromae* (Pat.) Griffon and Maubl [28]. have also been reported for causing diseases in *D. sissoo*.

Tree improvement programs in developing world have largely been focused on productivity improvement of timber and to a lesser extent on disease resistance [29]. Timber tree species like *D. sissoo* have a rotation age of 30 years or more which has also added to the problem of screening for disease resistance in the field despite widely reported infections and hence, necessitates finding a quicker and reliable system to identify disease-resistant genotypes of *D. sissoo*. Screening and selection of disease-resistant clones by *in vitro* co-culturing under gnotobiotic conditions of desired cloned genotypes with the fungal pathogen or toxin is an important crop improvement tool and has been adopted by different researchers [30–36]. *In vitro* growth condition is not only highly reproducible but also minimize the influence of external abiotic and biotic factors and ensures homogenous interaction between *in vitro* cultures of desirable host genotypes and inoculums of only known microbe giving a perfect gnotobiotic condition and thus, evincing a robust system to study plant-microbe interactions and facilitates estimation of resistance of desirable host plant genotypes [37, 38]. In the present study, callus and plantlets of ten genotypes of *D. sissoo* were co-cultured *in vitr*o along with virulent strains of *Fsd* in a dual culture setup. This in vitro screening of *D. sissoo* genotypes against virulent strains of *Fsd* was aimed at studying the variability and selection of resistant *D. sissoo* genotypes which, therefore, can be used as an indirect selection method for disease-resistant genotypes of tree species [39].

## Methods

### Plant material

Phenotypically superior trees of *D. sissoo* have been selected from various parts of northern India, cloned through branch cuttings are being maintained in clonal multiplication block. Ten genotypes that were part of long-term field trials for improved productivity were selected for this study (Table 1). Genotype 14, a commercial cultivar of *D. sissoo* resistant to shisham die-back was also chosen for the study [40].

**Table 1:** Source location of selected genotypes of *D. sissoo* in the study

Sl. No.	Genotype number	Source location		
		Locality	District	State
1.	10	Sabalgarh, Pathri	Haridwar	Uttarakhand
2.	14	Sabalgarh, Pathri	Haridwar	Uttarakhand
3.	19	Shah Mansurpur	Saharanpur	Uttar Pradesh
4.	24	Collectorbuck Ganj	Bareilly	Uttar Pradesh
5.	41	Hasanpur, Tulsipur	Gonda	Uttar Pradesh
6.	66	Chhachhrauli Range	Yamuna Nagar	Haryana
7.	201	Hasanpur, Tulsipur	Gonda	Uttar Pradesh
8.	204	Tulsipur	Gonda	Uttar Pradesh
9.	232	Birpur-Bhambar	Gonda	Uttar Pradesh
10.	237	Bankatwa	Gonda	Uttar Pradesh

### Fungal material

*F. solani* f. sp. *dalbergiae* strains (isolate no. 1145 and 1149) were procured from the National Type Culture Collection (NTCC) and were cultured in Petri dishes (9 cm) on potato dextrose agar (PDA) medium at 25 ± 2 °C.

### In vitro establishment of aseptic cultures of *D. sissoo*

#### Explant sterilization

Healthy and uninfected nodal segments of the selected genotypes of *D. sissoo* were used for aseptic culture establishment. Explants were washed in running tap water followed by soaking in a 0.5 % solution of antiseptic Cetrilak® (Cetrimide 5% w/v, India) and then in an aqueous solution of 0.1 % Bavistin® (Carbendazim WP; 50 % w/v, India). Surface sterilization of explants was carried out in a laminar airflow with 0.1% Mercuric chloride [41, 42].

#### Axillary bud proliferation and in vitro rooting

Nodal segments of the branch containing single axillary bud from each accession were collected, sterilized, and inoculated. For bud induction and multiplication, best-responded treatments, Murashige and Skoog (MS) medium [43] comprising 4.44 μM 6-Benzylaminopurine (BAP) + 2.69 μM 1-Naphthaleneacetic acid (NAA) and MS medium comprising 4.44 μM BAP + 1.34 μM NAA, were used, respectively [41]. Cultures were maintained on standardized multiplication medium at 5 weeks interval and repeated further in subsequent sub-culturing. Micro shoots of size > 2.5 cm were excised and transferred for root induction in half strength MS medium supplemented with Indole-3-butyric acid (IBA) at 4.92 μM concentrations [41]. Culture medium pH was adjusted to 5.8 and autoclaved for 15 min at 121 °C and 1.0 × 10^5^ Pa. Incubation temperature of culture room was 25 ± 2 °C and 55 ± 5 % relative humidity under a 16/8 hr (light/dark) photoperiod with light supplied by cool-white fluorescent tubes (Philips, India) at an intensity of 35 μmoles/m^2^/s.

#### Callus induction

Nodal explants of *D. sissoo* were collected from each genotype and sterilized as mentioned earlier, further inoculated on MS medium supplemented with BAP (2.22 - 6.66 μM) alone or in combination with 2,4-Dichlorophenoxyacetic acid (2,4-D; 2.26 - 6.79 μM) for callus induction. The callus was maintained on MS medium supplemented with 4.44 μM BAP and 2.69 μM NAA in culture condition as mentioned above for further in vitro screening [41].

### In vitro screening and selection of resistant *D. sissoo* genotypes against *Fsd*

#### Fungal inoculum preparation

A mycelial disc (4 mm dia) from growing margins of the *Fsd* culture was transferred to an Erlenmeyer flask (250 ml) containing 100 ml Carboxy Methyl Cellulose (CMC) medium [44] for sporulation. The culture was incubated for 15 days at 25 ± 2 °C and then viewed in a hemocytometer slide for conidial count. Consequential conidial suspension was diluted to the desired concentration (1 × 10^1^, 1 × 10^3^, and 1 × 10^5^ conidia/ml) in the appropriate inoculation medium.

#### Screening of callus against *Fsd*

Callus of each genotype of *D. sissoo* was inoculated on a standardized multiplication medium. On growing callus 5 μl droplet of conidial suspensions of *Fsd* (1145 and 1149) at three concentrations (1 × 10^1^, 1 × 10^3^ and 1 × 10^5^ conidia/ml) were inoculated atop the center of the callus and incubated at 25 °C ± 2 as described before. After inoculation of callus tissue with conidial suspensions of *Fsd*, the extent of infection was assessed on the 9th day by measuring the diameter of fungal growth on the tissue as well as conditions of callus, i.e., either dead or alive. Fungal growth (cm) was measured by taking the average of diameters at both X-X’ and Y-Y’axes of spread. Means of fungal radial diameter were analyzed using a parametric test.

#### Screening of plantlets against *Fsd*

In vitro rooted plantlets of each genotype of *D. sissoo*, were inoculated with *Fsd* (1145 and 1149) conidia at 1 × 10^5^ conidia/ml concentration in 0.1% water agar supplemented with MS salts, and incubated in culture conditions as described before. The plantlets were infected only with the highest *Fsd* conidial concentration as the differences during in vitro screening of callus of ten *D. sissoo* genotypes were notably evident at this concentration. The extent of infection was assessed at regular intervals throughout a 15-day period of incubation using the following disease scores: 0 = healthy plant, l = main root tip necrotic, 2 = whole root system infected, 3 = stem infected and appearance of wilt symptoms, 4 = whole plant wilted, and 5 = plant dead. Infection extent of each genotype was scored from 0 to 5 at the end of 5th, 7th, 9th, 11th, 13th, and 15th days. On each observed day, means of infection extent score of genotypes were compared using non-parametric test.

### Experimental design and statistical analyses

The experiments were laid in completely randomized design (CRD) with five replicates for each treatment and normal data were analyzed using analysis of variance (ANOVA) in Genstats 5 edition 3.2 for PC/Windows NT (Copyright 1995, LAWES Agricultural Trust (Rothamsted Experimental Station) and means were compared with least significant difference (LSD). Non-normal data analyzed using Kruskal-Wallis (KW) test in SPSS statistics-23. Results with a significant difference were KW test compared using rank corresponding to mean of infection extent of plantlet of genotype on each observed day. The genotype having rank with low numerical value were resistant whereas genotype having rank with high numerical were susceptible against the inoculated isolate of *Fsd*.

## Results

### In vitro response of callus of ten genotypes of *D. sissoo* against infection to *Fsd*

**Fig. 2:**
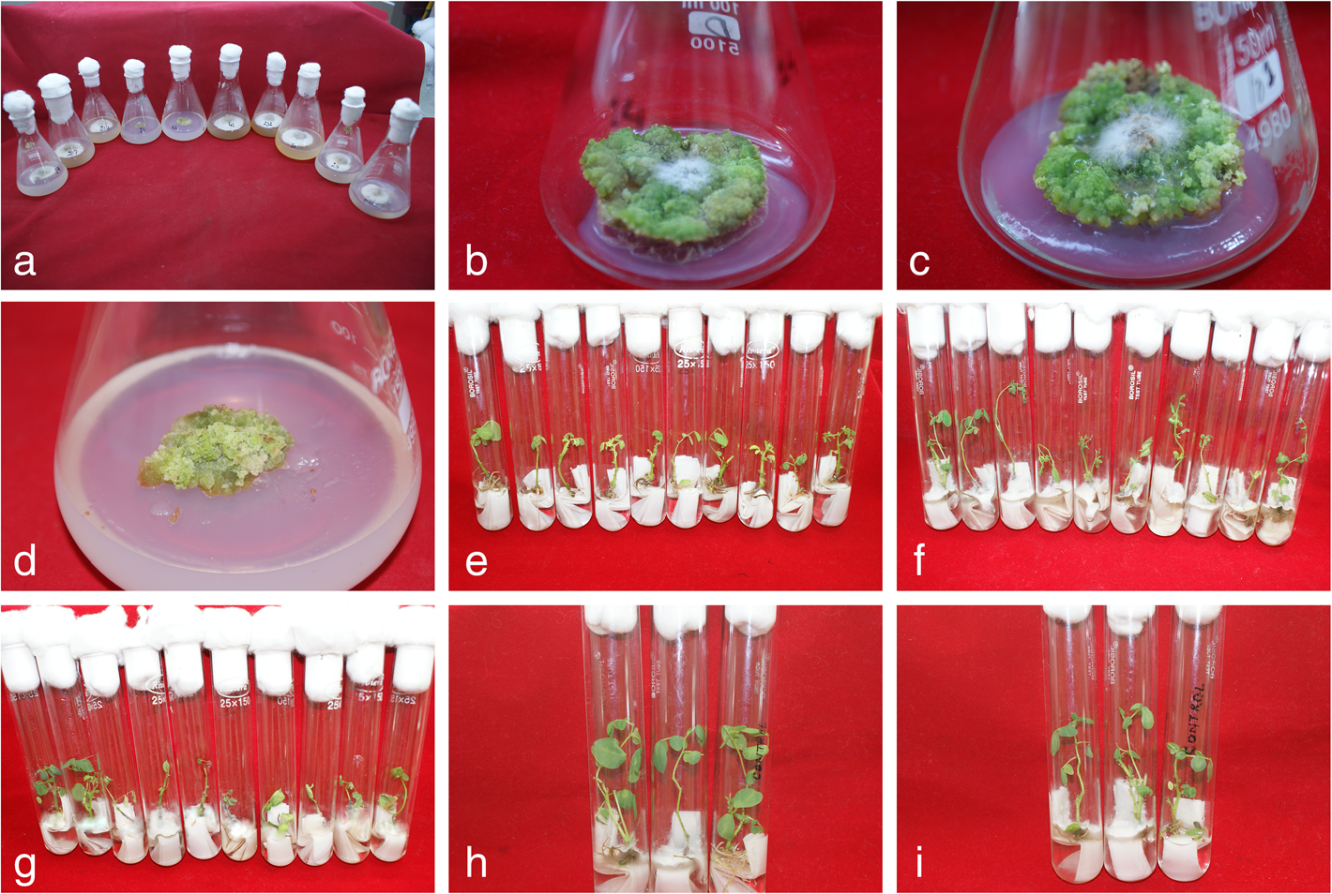
**a** Effect of *Fsd* isolate 1145 on in vitro raised callus of *D. sissoo* genotypes, from left to right genotype 19, 237, 204, 14, 66, 41, 232, 10, 24, and 20. **b** Callus of genotype 14 inoculated with *Fsd* (1149) after 30 days of infection. **c** Callus of genotype 66 inoculated with *Fsd* (1145) after 30 days of infection. **d** Control for (**a**). **e** Control for (**f** and **g**; from left to right genotypes 14, 66, 232, 237, 204, 24, 19, 41, 201 and 10). **f** Effect of *Fsd* isolate 1145 on in vitro raised plantlets of ten genotypes of *D. sissoo* (from left to right genotypes 14, 66, 232, 237, 204, 24, 19, 41, 201 and 10). **g** Effect of *Fsd* isolate 1149 on in vitro raised plantlets of ten genotypes of *D. sissoo* (from left to right genotypes 14, 66, 232, 237, 204, 24, 19, 41, 201 and 10). **h** Plantlets of genotype 14 infected with both isolates of *Fsd* 1149 (left most test tube) and 1145 (second test tube from left) and uninfected control (third test tube from left) after 3 weeks of infection. **i** Plantlets of genotype 66 infected with both isolates of *Fsd* 1149 (left most test tube) and 1145 (second test tube from left) and uninfected control (third test tube from left) after 3 weeks of infection

Results of the experiment suggest that *Fsd* isolates (1145 and 1149), *D. sissoo* genotypes, and the interaction between them had significantly affected the extent of fungal infection on the callus. High concentration (1 × 10^5^ conidia/ml) of both isolates resulted in maximum spread of fungus 3.12 cm and 3.14 cm, respectively, on callus after 9 days whereas low concentration (1 × 10^1^ conidia/ml) of both isolates resulted in minimum spread of fungus on callus 1.04 cm and 1.28 cm, respectively. Also, the extent of fungal infection on callus caused by each treatment differed significantly. Effect of *D. sissoo* genotypes on the growth of *Fsd* mycelium on callus was significant; fungal spread of both *Fsd* isolates 1145 and 1149 was least on callus of genotype 14 (0.9 cm and 1.16 cm, respectively) whereas genotypes 232 and 41 could not restrain the fungal spread of isolates 1145 and 1149, respectively, and thus, resulted in maximum spread of 2.90 cm and 2.89 cm, respectively. Fungal growth on callus of genotype 66 for *Fsd* isolates 1145 and 1149 was 1.00 cm and 1.23 cm, respectively, which is at par with genotype 14 (Table 2, Fig. 2a–d). Among the genotypes, callus of genotypes 14 and 66 showed resistance to fungal growth of both the *Fsd* isolates.

**Table 2:** Effect of conidial concentration of *Fsd* (1145 and 1149) on callus of *D. sissoo* genotypes

**Genotypes**	**Spore concentration**							
	**1 × 10**^**1**^	**1 × 10**^**3**^	**1 × 10**^**5**^	**Mean (cm)**	**1 × 10**^**1**^	**1 × 10**^**3**^	**1 × 10**^**5**^	**Mean (cm)**
	**Fungal diameter for isolate 1145 (cm)**				**Fungal diameter for isolate 1149 (cm)**			
14	0.8	0.8	1.1	0.9	1.1	1.1	1.3	1.2
66	0.9	0.9	1.3	1	1.1	1.1	1.5	1.2
19	0.9	1.3	2.4	1.5	1.2	1.6	2.6	1.8
41	1.0	1.9	3.0	2.9	1.2	2.0	3.0	2.1
24	1.0	1.8	3.4	2.1	1.3	2.0	3.6	2.3
10	1.1	2.2	3.9	2.4	1.4	2.3	3.8	2.5
201	0.9	1.1	2.2	1.4	1.2	1.5	2.6	1.7
204	1.2	2.1	4.2	2.5	1.4	2.3	4.0	2.6
232	1.4	2.4	5.0	2.9	1.5	2.5	4.7	2.9
237	1.3	2.3	4.78	2.8	1.5	2.4	4.5	2.8
**Mean (cm)**	1.04	1.68	3.12		1.28	1.88	3.14	
	**Variable**	**LSD at 5%**			**Variable**		**LSD at 5%**	
	Genotypes	0.20			Genotypes		0.13	
	Treatment	0.29			Treatment		0.17	
	Genotypes × treatment	0.34			Genotypes × treatment		0.22

Observations on the interaction between host genotypes and conidial concentrations of *Fsd* isolates (1145 and 1149) revealed that maximum spread of fungus was 5.02 cm and 4.68 cm, respectively, on callus of genotype 232 at 1 × 10^5^ conidia/ml concentration whereas a minimum spread of fungal growth 0.77 cm and 1.06 cm, respectively, was observed on callus of genotype 14 inoculated with 1 × 10^1^ conidia/ml concentration. An increase in *Fsd* mycelium spread was observed in callus of genotype 232 from 1.42 to 5.02 cm upon increasing conidial concentration of isolate 1145 from 1 × 10^1^ to 1× 10^5^ conidia/ml and similarly from 1.53 to 4.68 cm for isolate 1149. On the other hand, genotype 14 apparently restricted fungal growth of isolate 1145 on callus as no significant difference in fungal spread was observed when conidial concentration was increased from 1 × 10^1^ to 1 × 10^5^ conidia/ml. However, for isolate 1149 an increase in conidial concentration from 1 × 10^1^ conidia/ml to 1 × 10^5^ conidia/ml significantly affected the fungal spread on callus of genotype 14. It was interesting to note that the fungal spread on callus of genotype 14 was non-significant when concentration was increased from 1 × 10^1^ to 1 × 10^3^ conidia/ml as well as from 1× 10^3^ conidia/ml to 1× 10^5^ conidia/ml. Genotype 66, though not as promising as genotype 14, showed some resistance as fungal spread on callus was non-significant when conidial concentration of both isolates was increased from 1 × 10^1^ to 1 × 10^3^ conidia/ml, however fungal spread on callus differed significantly when conidial concentration of both isolates was increased from 1 × 10^3^ conidia/ml to 1 × 10^5^ conidia/ml. In other genotypes spread on callus differed significantly when conidial concentration of both isolates was increased from 1 × 10^1^ to 1 × 10^3^ conidia/ml as well as from 1 × 10^3^ to 1× 10^5^ conidia/ml (Table 2, Fig. 2a–d).

### In vitro response of plantlets of ten genotypes of *D. sissoo* against infection of *Fsd*

Plantlets of ten genotypes of *D. sissoo* were screened in vitro against two isolates of *Fsd* and observation was assessed using a disease score. Disease score data was non-normal so KW test was applied and significantly varying treatments were compared using rank. Results suggest that genotype 14 ranked first on all observed days except on day seven, where it was a joint second against isolate 1149. The corresponding mean disease score of genotype 14 on day five was 0.4 against both isolates which increased to 2.4 and 2.6 against isolates 1145 and 1149, respectively, on the 15th day. Genotype 66 ranked second consistently on all observed days against both isolates and its corresponding mean disease score was 0.6 on the 5th day which grew to 2.8 on the 15th day against both isolates. Genotype 19 managed rank 3.5 and 3.0 against 1145 and 1149, respectively, on the 5th day, 3.0 against both isolates on the 9th day, 11th day, and 13th day but rank 6.5 on the 15th day. The corresponding mean disease score was 0.8 on day 5th which steeply reached to 5.0, which meant completely dead plantlets, on the 15th day, which suggests that slight resistance was shown by genotype during initial days of treatment but was lost by the 15th day. Similar results were obtained for genotype 41.

Plantlets of susceptible genotypes were prone to infection from the beginning and maintained susceptibility throughout the observation. Genotypes 10, 24, 201, 204, 232, and 237 on the 5th day had mean disease score more than one and their relative ranks were 6, 8.5, 6.0, 10, 6, and 8.5, respectively, against isolates 1145 whereas against isolate 1149 their ranks were 5, 8, 8, 8, 8 and 8, respectively. Five genotypes 24, 201, 204, 232, and 237 scored more than three against isolate 1149 on 7th day and their corresponding ranks were 8, 8, 8, 8, and 10, respectively, though against isolate 1145 genotypes 10, 24, 201, 204, 232, and 237 had mean disease scores of 1.8, 2.0, 1.0, 1.8, 1.0, and 2.4, respectively, and the corresponding ranks were 7.5, 9, 4, 7.5, 4, and 10, respectively. On the 11th day, genotypes 24, 232, and 237 had the highest mean disease scores of 5 and the corresponding ranks were 9 against isolate 1149 whereas the same genotypes had mean disease scores of 4, 4, and 4.2, respectively, with the corresponding ranks 8.5, 8.5, and 10, respectively, against isolates 1145. Genotypes 10, 24, 201, 204, 232, and 237 had a mean disease score of 5 on the 13th and 15th day against both isolates (Table 3, Fig. 2e–i).

**Table 3:** Disease scoring of plantlets of ten *D. sissoo* genotypes inoculated with *Fsd* (1145 and 1149)

	5th day				7th day				9th day				11th day				13th day				15th day			
Isolate no.	1145		1149		1145		1149		1145		1149		1145		1149		1145		1149		1145		1149	
Genotype no.	Mean^a^	Rank^b^	Mean^a^	Rank^b^	Mean^a^	Rank^b^	Mean^a^	Rank^b^	Mean^a^	Rank^b^	Mean^a^	Rank^b^	Mean^a^	Rank^b^	Mean^a^	Rank^b^	Mean^a^	Rank^b^	Mean^a^	Rank^b^	Mean^a^	Rank^b^	Mean^a^	Rank^b^
**10**	1	6	1.8	5	1.8	7.5	2.6	5	2.8	5.5	3.8	6	3.8	6.0	4.8	6	5.0	7.5	5	7	5.0	6.5	5	6.5
**14**	0.4	1	0.4	1	0.6	1.0	1	2	1.0	1.0	1.6	1	1.6	1.0	1.8	1	2.2	1.0	1.8	1	2.4	1.0	2.6	1.0
**19**	0.8	3.5	0.8	3	1.2	6.0	2	4	1.6	3.0	2.6	3	2.8	3.0	3.8	3	3.6	3.0	4.8	3	5.0	6.5	5	6.5
**24**	1.2	8.5	2	8	2.0	9.0	3	8	3.0	7.5	3	5	4.0	8.5	5	9	5.0	7.5	5	7	5.0	6.5	5	6.5
**41**	0.8	3.5	1	4	1.0	4.0	2	4	1.8	4.0	2.8	4	3.0	4.0	4	4	3.8	4.0	5	7	5.0	6.5	5	6.5
**66**	0.6	2	0.6	2	0.8	2.0	1	2	1.2	2.0	2	2	1.8	2.0	2.4	2	2.6	2.0	2.6	2	2.8	2.0	2.8	2.0
**201**	1.0	6	2	8	1.0	4.0	3	8	2.8	5.5	4	8	3.8	6.0	4.8	6	5.0	7.5	5	7	5.0	6.5	5	6.5
**204**	1.4	10	2	8	1.8	7.5	3	8	3.0	7.5	4	8	3.8	6.0	4.8	6	5.0	7.5	5	7	5.0	6.5	5	6.5
**232**	1.0	6	2	8	1.0	4.0	3	8	3.2	9.5	4	8	4.0	8.5	5	9	5.0	7.5	5	7	5.0	6.5	5	6.5
**237**	1.2	8.5	2	8	2.4	10.0	3.2	10	3.2	9.5	4.2	10	4.2	10.0	5	9	5.0	7.5	5	7	5.0	6.5	5	6.5

^a^Mean disease score reflects disease severity and ^b^rank reflects the ability to resist *Fsd* infection

## Discussion

Vascular wilt, blight, bakanae disease, etc. caused by *Fusarium* species have been reported for widespread plant mortality [44–47]. Selection of disease-resistant plant varieties through in vitro screening has been utilized in the improvement of crops against *Fusarium* spp, viz. strawberry [30], *Musa* spp [33], wheat [48, 49], date palm [50], alfalfa plants [51], and passion fruit [52].

This study is the first report of in vitro screening and selection of resistant *D. sissoo* genotypes against *Fsd* vascular wilt. Among ten genotypes, it can be concluded that the callus of genotype 14 has resisted the fungal infection, followed by genotype 66. But in genotype 66, the resistance diminished gradually with an increase in conidial concentration to 1 × 10^5^ conidial/ml. Similar studies of callus-fungal interactions for disease resistance selection have been reported in woody species like *Acacia pulchella*, *Eucalyptus calophylla*, *E. marginata* [31], *Pinus eschinata* and *P. virginian*a [53], *Prunus persica* [54], *Pinus ellottii* [55], *Citrus sinensis* and *C. limon* [56], *Fagus sylvatica* [57], *Pinus nigra* and *P. sylvestris* [58], and *Malus domestica* [59].

In vitro cloned plantlets of *D. sissoo* genotypes infected under in vitro condition with conidial suspension of *Fsd* showed results similar to callus. After infection, it was observed that fungal mycelium grew rapidly and a cottony mass of mycelium could be seen around the rhizosphere of plantlets, which may be due to humid conditions of the culture vessels providing a favorable environment for mycelial growth [60]. Nonetheless, on the 15th day of observation, plantlets of genotypes 14 and 66 had a significantly lesser disease severity index (between 2 to 3) implying that initial symptoms of wilt appeared after fungal growth around the rhizosphere but the plantlets remained green, healthy, and had fewer necrotic lesions in the roots whereas plantlets of remaining eight genotypes completely wilted and died. Similar findings have been reported for other plantlet-microbe interactions [33, 51, 53, 61–65].

In vitro screening of clonal host genotypes against specific strains of the pathogen in a dual culture setup is a perfect system for gnotobiotic studies in plant-microbe interaction and it gives an opportunity to estimate the resistance or susceptibility of clones of host plant (*D. sissoo* genotypes) by ensuring only one microbe (*Fsd* isolate in this case) is infecting only one host genotype in a culture flask in axenic condition with growth environment. Plantlets with complete root and shoot system or callus (mostly representing the unorganized cellular growth) both of the same genotype showed a similar trend. Growth condition and nutrient rich MS growing media favor the growth of microbe rather than the host in this dual culture setup and thus ensuring strict criteria for selection of resistant genotypes.

## Conclusions

The study, thus, concludes, that callus of two genotypes of *D. sissoo* (14 and 66) showed resistance against *Fsd* under in vitro conditions whereas the remaining eight genotypes were susceptible. Similar results were observed for in vitro screening of plantlets of *D. sissoo* genotypes against *Fsd*. This suggests that in vitro screening of candidate genotypes of *D. sissoo* against *Fsd* under gnotobiotic conditions may be an effective as well as a quick method for screening and selection of disease-resistant genotypes. Moreover, by this method, a large number of *D. sissoo* genotypes could be screened in limited time and space, hence, assisting in the process of screening and selection of disease-resistant genotypes.

## Data Availability

Not applicable.

## Abbreviations

PDA: Potato dextrose agar
MS: Murashige and Skoog basal media
BAP: 6-Benzylaminopurine
NAA: 1-Naphthaleneacetic acid
IBA: Indole-3-butyric acid
2,4-D: 2,4-Dichlorophenoxyacetic acid
CMC: Carboxymethyl cellulose
*Fsd*: *Fusarium solani* f. sp. *dalbergiae*
CRD: Completely randomized design
ANOVA: Analysis of variance
LSD: Least significant difference
KW: Kruskal-Wallis test
